# Exploring the Potential of Anticipated Regret as an Emotional Cue to Improve Bowel Cancer Screening Uptake

**DOI:** 10.1155/2017/2949020

**Published:** 2017-02-02

**Authors:** Ian T. Zajac, Amy Duncan, Suzana Freegard, Carlene Wilson, Ingrid Flight, Deborah Turnbull

**Affiliations:** ^1^Health & Biosecurity, Commonwealth Scientific & Industrial Research Organisation, Adelaide, SA, Australia; ^2^School of Psychology, University of Adelaide, Adelaide, SA, Australia; ^3^Discipline of General Practice, Flinders University, Adelaide, SA, Australia; ^4^Flinders Centre for Innovation in Cancer, Flinders University, Adelaide, SA, Australia; ^5^Cancer Council South Australia, Eastwood, SA, Australia

## Abstract

*Objective*. Bowel cancer is currently the second leading cause of cancer-related death in Australia and screening participation is suboptimal. This study examined the role of emotion in the form of anticipated regret (AR) and its relationship to screening intentions.* Methods*. *N* = 173 persons aged 45 to 80 years completed a survey measuring demographic variables, readiness to screen, relative importance of health by comparison to other life priorities, satisfaction with current health, and AR if not participating in future bowel cancer screening.* Results*. AR was a significant predictor of future screening intentions. Those with higher levels of AR were seven times more likely (OR = 7.18) to intend to screen in the future compared to those with lower AR. This relationship was not compromised when controlling for other variables including gender and satisfaction with one's health. AR levels were significantly lower in people who had been screened previously and in those with full health insurance.* Conclusions*. These results demonstrate that AR is uniquely related to future bowel cancer screening intentions. Future studies should continue to consider this as a useful target for behavioural interventions and identify new ways of delivering these interventions to improve their reach.

## 1. Background

Colorectal cancer (CRC) is the second most common cause of cancer-related death in Australia [1]. Treatment success ranges from 90% for cancers detected early to 10% for late stage metastatic cancer [2]. Symptoms of CRC generally only become apparent during later stages of the disease when prognosis is poor. Thus, early detection of CRC via screening is critical to improving outcomes [3, 4].

In Australia, the National Bowel Cancer Screening Program (NBCSP) offers a free home stool screening test to people aged 50, 55, 60, 65, and 74 years, initially at five yearly intervals. Biennial screening will become available for all Australians from age 50 onwards by 2019. The home stool test requires small samples to be collected from two consecutive bowel movements. Regular screening in people aged 50 years and over has been shown to significantly reduce CRC incidence and mortality by 25% in randomised controlled trials [4]. However, despite the efficacy and low cost of wide scale screening, participation in these offers is low and declining. In the first year of the pilot program (2006-2007) participation was 41% [5] dropping down to 33.4% in 2012-2013 [6]. Given that in the same period the screening rate was 60% for breast cancer [7] and 58% for cervical cancer [8], it is not unreasonable to expect that an increase in bowel screening rates can be achieved. Identifying new targets for public health interventions is critical to improving early detection and reducing CRC-related mortality.

Theoretical models from the health and social psychology literature provide insight into the reasons for poor screening uptake and inform interventions aimed at improving this behaviour. The theory of planned behaviour (TPB) holds that an individual's execution of a behaviour is determined by their intention to engage in the behaviour and perceived behavioural difficulty, including their perception of the difficulty of performing the behaviour and their belief that the behaviour is within their control [9]. Other models build on this by explaining the factors that determine intention as well as perceived behavioural difficulty.

Social cognitive models have, however, been criticised for a reliance on cognitive variables and the underlying assumption that individuals make decisions logically and rationally, to the exclusion of any consideration of the role of emotion in human decision-making [10]. The addition of measures such as “anticipated regret” to these models addresses this criticism by accounting for a part of the affective aspect of behavioural choices. Regret itself is a negative emotion resulting from thinking that a current situation would have been better had we acted differently in the past. Regret about not acting can be anticipated and prompting this anticipation in individuals currently considering whether or not to screen can prompt the behaviour as a way to avoid regret in the future [11].

A meta-analysis exploring anticipated regret as an additional predictor in the TPB demonstrated that anticipated regret significantly adds to the prediction of both intentions and behaviour after variables from the traditional TPB model have been accounted for [10]. This was shown to be the case for a variety of different health behaviours including condom use and junk food, alcohol, and tobacco consumption. A more recent meta-analysis supported these results, showing again that AR was moderately strongly linked to intentions and also behaviour but to a slightly weaker extent [12]. In the context of cancer screening, a recent study demonstrated that encouraging women to indicate their level of anticipated regret if they did not participate in a cervical screening opportunity (i.e., “If I did not attend for a cervical smear in the next few weeks, I would later wish I had”) substantially increased participation when compared to those who were asked to reflect only on the traditional TPB variables [13].

With regard to bowel cancer screening, one study has attempted to utilise this construct to increase compliance. The study asked people invited to screen for CRC to complete a set of questions that required them to anticipate regret associated with nonparticipation in their current screening offer. Although there was no effect of the intervention at the intention-to-treat level, the intervention significantly increased screening in those who had lower intentions to screen [11]. This result highlights the importance of developing an intervention that is salient for all, not just those willing to complete and return a behavioural survey.

Finding effective targets for use in behavioural interventions aimed at improving CRC screening participation is an important goal. A critical step in this process is to first identify that a relationship exists between emotional drivers of behaviour—such as anticipated regret—and the target behaviour of interest; in this case, bowel cancer screening. Therefore, the purpose of this study was to investigate the association between anticipated regret and future screening intentions in the target population cross-sectionally, and to test the robustness of any relationship in the presence of other predictors of screening including demographics, and prior screening participation. The study also sought to explore whether demographic factors explain differences in anticipated regret in an Australian population.

## 2. Methods

### 2.1. Participants and Recruitment

Participants (*N* = 173; 65% male) between the ages of 45 and 80 living in South Australia volunteered to complete an online survey. Recruitment occurred via advertisements in the University of Adelaide electronic newsletter and promotion on social media accounts of the University of Adelaide and the Commonwealth Scientific and Industrial Research Organisation (CSIRO). Emailed invitations were also sent to men registered with the Freemasons Foundation Centre for Men's Health, University of Adelaide, as being interested in participating in research projects. Potential participants were invited to complete an online survey about screening for bowel cancer and were also encouraged to share the survey link or advertisement with their own contacts (i.e., snowball sampling).

A study information form accompanied the survey and participants provided consent to participate online before commencing the survey phase. All participants who completed the survey were given the option to enter a prize draw to win one of two tablet computers valued at approximately $380 Australian dollars. Survey responses were analysed in anonymous form; personal contact information provided for the prize draw was not downloaded alongside survey responses. The study was approved by the human research ethics committee at the School of Psychology, the University of Adelaide. Recruitment commenced in October of 2014 and the survey was available for completion until December 2014.

### 2.2. Survey Measures

*Demographic Information and Prior Screening Experience*. Participants were asked to provide demographic information (age, sex, marital status, highest level of education, and employment status) as well as their level of private health insurance coverage (none, only hospital cover, hospital and ancillary cover, and both hospital and ancillary cover). In addition to this, participants' readiness to participate in home stool screening was measured utilising a staging algorithm [14] consistent with the Precaution Adoption Process Model (PAPM, [15]). Questions determined if respondents had heard of home stool screening prior to participating in this study (yes or no) and their intentions to screen in the future (yes, no, or unsure). Participants were also asked if they had previously completed a home stool test.

*Anticipated Regret*. Anticipated regret was measured with two items previously utilised in colorectal cancer screening research [16]. Items were as follows: (1) if I were to receive an offer to screen for colorectal cancer and chose not to complete it, I would later feel regret and (2) if I were to receive an offer to screen for colorectal cancer and chose not to complete it, I would later wish I had. Responses were measured on a five-point Likert scale ranging from strongly disagree (1) to strongly agree (5). 

*Health Priorities*. In order to measure how highly participants regarded their health relative to other factors, they were provided with a list of ten “life priorities” [17] from which they were asked to select the three most important factors and rank them from one (most important) to three. The ten options to choose from included the participants' own health, the health of loved ones, their ability to work or find work, relationships, the environment, finances and standard of living, education and social life. Participants were also asked to rate their satisfaction with their overall health on a scale of one (no satisfaction at all) to eleven (completely satisfied).

### 2.3. Power and Statistical Analyses

Given the broad aim of assessing the relationship between anticipated regret and future screening intentions, a power analysis was conducted using G*∗*Power software [18]. In order to identify a weak relationship (*R*^2^ = 0.15) between up to six independent predictors and future screening intentions in a single multiple regression model, it was determined that at least *N* = 125 participants were required for adequate power (*β* = 0.95; *α* ≤ 0.05). The present sample exceeded this requirement. In regard to the survey completion rate, a total of *N* = 198 commenced the online survey, but *N* = 23 dropped out early on, providing less than 65% complete data. Two participants were also removed because they resided outside of South Australia. The final survey completion rate was 87% (173/198).

Scores on anticipated regret (AR) were determined by averaging the two items; these items were strongly correlated (*r* = 0.85, *p* < 0.001). Participants were split into two groups, lower AR (average Likert response ≤ 4) and higher AR categories (response = 5) because the distribution was strongly negatively skewed. Adjustments were also made to other variables to maintain relative balance across group categories where there were discrepancies between numbers per response category, or to deal with skewness. These adjustments were as follows: (1) health satisfaction responses were recoded into moderately satisfied (M = 4.54, SD = 1.53), satisfied (M = 7.52, SD = 0.50), and completely satisfied groups (M = 9.21, SD = 0.41); (2) participants were coded as ranking their health as their first priority, their second priority, or their third/not-at-all priority; (3) future screening intentions were recoded into unsure/no-intention and intend-to-screen groups; (4) health insurance was categorised as fully insured or partial/no insurance; and (4) for age, participants were organised into 45 to 54 years, 55 to 64 years, and 65 years and over groups. Only nine participants indicated that they had never heard of home stool screening and, given disparate group sizes, this variable was not used for analyses. Descriptive statistics were used to examine the overall characteristics of the survey sample according to the various groupings. Binary logistic regressions were used to assess the association between predictor variables and future intentions to use a home stool test and level of AR.

## 3. Results

### 3.1. Sample Characteristics

Sample characteristics are provided in Table 1. The number of individuals within each variable grouping was adequate for analysis. There were around twice as many males than females in this study and participants tended to be married. Most participants reported that they had full private health coverage and the majority of participants indicated that they wished to screen for bowel cancer using a home stool test in the future.

### 3.2. Anticipated Regret and Future Screening Intentions

The associations between the predictor variables, AR, demographic variables, and health priorities and the outcome future screening intention are displayed in Table 1. There is a very marked association between AR and future screening intention, with individuals in the higher AR category around seven times more likely to indicate that they definitely wanted to screen compared to those with lower AR. The only other variable to predict future intentions in univariate models was prior screening use. Contrary to expectations, prior screeners were significantly less likely to want to screen in the future.

A multivariate model was built by entering all univariate predictors shown in Table 1 into a single multivariate model. This model specified entry and exit criteria for predictor variables of *p* ≤ 0.10 and *p* ≥ 0.15, respectively. As can be seen in Table 2, the only variables to emerge as multivariate predictors included sex, prior screening use, and health satisfaction. Women were almost four times more likely to want to screen in the future than men, whereas individuals completely satisfied with their health were less likely to want to screen compared to those moderately satisfied. AR again emerged as a very strong predictor of future screening intentions. Given that there were a large number of men in the present study as well as the unusual finding of an inverse relationship between prior screening and future screening intentions, model 2 included a sex *x* prior screening use interaction term, which was highly significant. To decompose this interaction, the relationship between prior screening use and future screening intentions was examined separately for males and females. This demonstrated that wanting to screen in the future was not related to prior screening in women (*β* = −0.11, SE = 0.86, odds ratio = 0.90, 95% CI = 0.17–4.84), but it was significantly inversely related to prior screening in men (*β* = −2.54, SE = 0.57, odds ratio = 0.08, 95% CI = 0.03–0.24).

### 3.3. Differences in Anticipated Regret

To assess the extent to which AR differed across individuals, a series of univariate and multivariate regression models were conducted. The results of these are shown in Table 3. As can be seen, AR was relatively robust in terms of its consistency across the various groups. However, at the univariate level, those who had screened previously were much less likely to indicate higher levels of AR. Additionally, those aged 65 and over were significantly more likely to have higher AR than the 45- to 54-year-olds. In the multivariate conditional model, the relationship between prior screening and AR remained, and the only other significant relationship concerned private health insurance. Specifically, those with full health insurance were likely to have lower levels of AR.

## 4. Discussion

The primary purpose of this study was to explore anticipated regret (AR) and its relationship to future bowel cancer screening intentions in an Australian sample. Our aim was to establish whether AR might be a useful target for behavioural interventions designed to improve bowel screening compliance in an Australian population. A secondary aim was to examine whether AR differed across individuals to determine whether it should be targeted in specific groups as opposed to all individuals.

The results of this study provide strong evidence that AR is an important predictor of intention to screen for bowel cancer and is in line with the results of previous research showing some association between AR and actual bowel screening behaviour in select groups of participants [11]. This emotion-based, behavioural driver was equally predictive of screening intentions as prior screening experience in the univariate regressions. In the multivariate models it was again strongly related to future intentions and comparable to prior screening behaviour. Both of these variables were more predictive of future intentions than gender and health satisfaction. In regard to the extent to which AR differed between individuals, the univariate models showed that prior screening use was related to lower AR, whilst those aged over 65 years had higher AR levels. In the multivariate model, prior screening use and health insurance were both associated with lower AR whilst age was no longer significant. In this study, the strength of the effect of AR on future intentions was quite large. This is consistent with other reviews showing strong links between this construct and intentions and although AR is promising as a target for behavioural interventions, the strength of its association with actual behaviour will likely be lower [10, 12].

The finding that prior participation in screening corresponded to lower AR is an interesting result. Elsewhere, emotional constructs including cancer worries have been linked to bowel cancer screening [19] and participation in screening is likely to alleviate these concerns. Thus, it is plausible that participants in the present study are less concerned about having bowel cancer given prior participation in screening and by extension, lower AR, because they consider it unlikely that they might have undetected cancer.

Participants who had used a home stool test previously were also significantly less likely to want to screen in the future. This is an unusual finding, with prior participation usually positively related to future screening behaviour [20]. An interaction between sex and prior screening showed that this relationship was only true for men, who are typically less likely to screen than women [21]. The present sample may have consisted of men who were particularly biased against future home stool screening and because of this, they would not regret not participating in the future. Alternatively, men may find the screening process more distasteful than women or may not understand the need for repeat screening, hence the impact of prior screening on future intentions. Further research is necessary to explore these results in more detail.

Given the robust relationship between AR and future intentions, this is likely to be a useful target variable for behavioural interventions. Appealing to this feeling may increase behavioural intention by raising the salience of the decision, which subsequently stimulates action [10]. For cervical screening, requiring individuals to answer questions concerning AR improved screening uptake [13]. For bowel cancer screening, the same approach demonstrated an effect of AR on screening uptake but only in individuals with low initial screening intentions who completed the questionnaire [11]. Further secondary analysis of the same study showed that AR was the only significant predictor of test uptake amongst this group, after controlling for other emotional variables such as disgust [22]. Neither of these randomised trials demonstrated any effect of the AR intervention at the intention-to-treat level and it is plausible that the absence of significant results in the abovementioned studies is a result of intervention design. The AR intervention in those studies was only completed by those individuals who actually took the time to answer optional questions which prompted participants to think about future regret they might experience if they did not screen. Thus, those who did not complete and return the questionnaire are unlikely to have experienced the intervention because they were not made to actively think about their potential AR, a point also highlighted by O'Carroll et al. [11]. An alternate and possibly more effective approach might focus on delivering a visually appealing brochure that provides emotional AR messages but does not require “actioning,” as is required by a set of questions measuring AR. Similar approaches to mass-messaging have been used previously to promote smoking cessation with the most effective messages being those concerned with subsequent illness and negative impacts of not quitting [23]. A comparable approach utilising paper-based messaging that emphasises the potential future negative impacts of undetected bowel cancer that may follow from not screening might be effective at improving screening uptake when delivered in combination with the test kit. However, there may be ethical issues associated with promoting negative emotional AR messages in a mass-messaging approach which need careful consideration, and there is currently no evidence of the use of this approach for this specific target (AR).

Whilst this study highlights an important area for further exploration there are some limitations to consider. Firstly, the study does not use a well-defined population based sampling frame. Data were obtained from a larger survey designed to explore the potential for different modes of delivery of bowel cancer screening information to improve screening uptake. It is possible, therefore, that those who volunteered for the study were interested in alternate forms of screening, explaining the unexpected inverse relationship between previous use of the test and intentions for future use. Similarly, as already discussed, the uneven gender distribution may also have influenced results. In this study there were more male responders than females responders and this may reflect the recruitment strategy which included distributing the study invitation to males involved with organisations including the Freemasons Foundation Centre for Men's Health in South Australia. Whilst there were no observed gender differences for levels of anticipated regret, it would be advisable to replicate the research in a balanced sample in order to confirm the extent to which anticipated regret and prior test use predict future screening in the general population.

Self-selection bias may also have influenced these findings. It has been noted that previous studies have only shown an association between AR messaging and actual behaviour in individuals who have actively engaged in those interventions by completing AR questionnaires. Thus, the association between AR and behavioural intentions shown herein may not be robust in those who did not engage with our study. It will be important for future studies to attempt to circumvent this bias and test the efficacy of AR messaging in the broader target population.

## 5. Conclusion

This study offers some promise for a new psychological target for health promotion messages designed to encourage uptake of bowel cancer screening. The cross-sectional design has limitations in comparison to randomised control trials that have targeted this variable [11], but the size of the effect supports that this variable may be a useful target in future trials in the Australian population. As organised screening programs continue to roll out and mature, it will become increasingly important for messages to evolve so as to retain public attention as well as to engage underscreened populations.

## Figures and Tables

**Table 1:** Sample characteristics and their univariate relationships with future screening intentions.

Predictor variable	Variable levels	Sample characteristics	Intending to use HST in the future (yes)^a^		Univariate effect size^b^
		% (*n*)	Odds ratio	95% CI	
Intends to use HST in the future	Unsure/no	16% (28)	N/A		
	Yes	84% (145)			

Anticipated regret	Lower	52% (90)	1	—	0.16
	Higher	48% (83)	7.18^*∗∗∗*^	2.37, 21.75	

Previous HST use	No	62% (108)	1	—	0.15
	Yes	38% (65)	0.18^*∗∗∗*^	0.08, 0.44	

Sex	Male	65% (112)	1	—	0.08
	Female	35% (61)	2.24	0.86, 5.87	

Age Cat	45 to 54 years	38% (65)	1	—	0.01
	55 to 64 years	29% (51)	1.42	0.52, 3.92	
	65 and over	33% (57)	1.21	0.47, 3.12	

Relationship status	Single	19% (32)	1	—	0.02
	Married	81% (141)	2.01	0.78, 5.11	

Health insurance	Partial/no insurance	27% (46)	1	—	0.01
	Fully insured	73% (127)	0.72	0.27, 1.89	

Employment status	Full-time employed	47% (81)	1	—	0.04
	Retired	27% (47)	1.95	0.72, 5.33	
	Part-time/other	26% (45)	2.93	0.93, 9.27	

Highest education	School/trade certificate	35% (61)	1	—	0.01
	Diploma/undergraduate	32% (56)	0.62	0.23, 1.67	
	Graduate diploma/postgraduate	32% (56)	0.79	0.28, 2.21	

Health ranking	Ranked 1st	33% (57)	1	—	0.03
	Ranked 2nd	30% (50)	0.39	0.21, 1.85	
	Ranked 3rd/not at all	38% (66)	0.4	0.14, 1.09	

Health satisfaction	Moderately satisfied	37% (64)	1	—	0.01
	Satisfied	49% (85)	0.76	0.31, 1.88	
	Completely satisfied	14% (24)	0.81	0.23, 2.95	

^a^Reference category for outcome is unsure/no. ^b^Nagelkerke effect size. HST = home stool test. ^*∗∗∗*^*p* < 0.001.

**Table 2:** Multivariate predictors of future screening intentions.

Model	Predictor variables	Levels	Intending to use HST in the future (YES)^a^	
			Odds ratio	95% CI
1	Anticipated regret	Lower	1	—
		Higher	7.28^*∗∗∗*^	2.12, 23.97
	Sex	Male	1	—
		Female	3.67^*∗*^	1.23, 10.97
	Previous HST use	No	1	—
		Yes	0.12^*∗∗∗*^	0.04, 0.36
	Health satisfaction	Moderately satisfied	1	—
		Satisfied	0.3^*∗*^	0.10, 0.91
		Completely satisfied	0.45	0.10, 2.13

2	Anticipated regret	Lower	1	—
		Higher	7.2^*∗∗*^	2.10, 24.70
	Sex	Male	1	—
		Female	0.8	0.17, 3.82
	Previous HST use	No	1	—
		Yes	0.062^*∗∗∗*^	0.02, 0.23
	Health satisfaction	Moderately satisfied	1	—
		Satisfied	0.27^*∗*^	0.08, 0.87
		Completely satisfied	0.47	0.10, 2.36
	Sex *x* previous HST	N/A	11.49^*∗∗*^	1.34, 98.30

^a^Reference category for outcome is unsure/no. ^*∗*^*p* < 0.05; ^*∗∗*^*p* < 0.01; ^*∗∗∗*^*p* < 0.001.

HST = home stool test.

Model 1: −2 log likelihood = 114.09, Nagelkerke *R*^2^ = 0.34.

Model 2: −2 log likelihood = 109.22, Nagelkerke *R*^2^ = 0.38.

**Table 3:** Relationships between sample characteristics and level of anticipated regret.

Predictor	Reference category	Higher anticipated regret^a^	
		Odds ratios	CI (95%)
*Univariate models*			
Previously used HST	No previous use	0.44^*∗∗*^	0.23, 0.83
Full health insurance	Partial/no insurance	0.56	0.28, 1.10
Female	Male	0.79	0.42, 1.49
55 to 64 years	45 to 54 years	0.85	0.40, 1.80
65 and over		2.10^*∗*^	1.02, 4.34
Married	Single	1.23	0.57, 2.67
Retired	Full-time employed	2.07	1.00, 4.29
Part-time/other		1.75	0.84, 3.65
Diploma/undergraduate	School/trade certificate	1.36	0.66, 2.82
Graduate diploma/postgraduate		0.95	0.46, 1.97
Ranked 2nd	Ranked 1st	1.38	0.66, 2.90
Ranked 3rd/not at all		0.58	0.28, 1.20
Satisfied with health	Moderately satisfied	1.47	0.77, 2.83
Completely satisfied with health		1.37	0.53, 3.51
*Multivariate predictors*			
Previously used HST	No previous use	0.40^*∗∗*^	0.21, 0.77
Full health insurance	Partial/no insurance	0.49^*∗*^	0.24, 0.99

^a^Reference category for outcome is lower anticipated regret. ^*∗*^*p* < 0.05; ^*∗∗*^*p* < 0.01.

HST = home stool test.
